# 

**Published:** 1870-09

**Authors:** 


### CONTENTS.

ORIGINAL COMMUNICATIONS.

Report on Dental Chemistry, ... 373
How Shall we best Impart Dental Instruction to Patients, ... 376
Society Discussions, ... 379

PROCEEDINGS OF SOCIETIES.

Discussions Before the Twentieth Annual Meeting of the Indiana State Dental Association, June 29th, 1870, ... 382
New Dental Society, ... 398

SELECTIONS.

Reproduction of Bone, ... 399
Annual Address before the Dental Society of New York, ... 402

CORRESPONDENCE, ... 413

EDITORIAL.

Chloroform Again, ... 415
Meeting of the Ohio State Dental Society, ... 416

